# The Hospital Nursing Supplement

**Published:** 1892-10-15

**Authors:** 


"The Hospital" Nursing Mirror.

Being the Extra Nursing Supplement of "The Hospital" Newspaper.

[Contributions for this Supplement should be addressed to the Editor, The Hospital, 140, Strand, London, W.C., and should have the word "Nursing" plainly written in left-hand top corner of the envelope.]

### En Passant.

ROYAL NATIONAL PENSION FUND FOR NURSES. —The *Glasgow Evening News* says that Miss Ross, the Lady Superintendent of the Victoria Infirmary, Glasgow, has been elected a member of the Council of this Fund, and she will be glad to give information and assistance to any nurse on personal application.

PROGRESS AT PRESTON.—The Board of Guardians have appointed a female nurse as superintendent at Preston Infirmary at a salary of £40 per annum. They have also decided to have an additional certified nurse and a probationer, both to be under the supervision of Miss Watkinson. This lady had held the post of assistant nurse for two years previously, and had given general satisfaction.

BANTRY BOARD AND ITS FEVER NURSE.—The Guardians having been advised by Dr. Popham to get a skilled permanent nurse for their Fever Hospital, have decided to advertise for one at the magnificent salary of £15 per annum. They must have original ideas of the amount of skill necessary for sick nursing, if they hope to obtain it at that rate. The same issue of the *Cork Herald* reports incidentally the case of a porter in the employ of the Guardians having taken action against an insane inmate who "threatened him." The prosecution was successful, the unfortunate lunatic, named Michael Sullivan, being sentenced to one month's imprisonment. If this is the way patients suffering fnom brain disease are treated at Bantry, we need not wonder that a £15 nurse (?) is deemed good enough for fever cases.

WORKHOUSE NURSING AT PEMBURY.—The sick poor have found an able champion in Sir Julian Goldsmid, and he does not believe in paupers being neglected either by night or day. The Tonbridge Board of Guardians received the report of the committee, which has been inquiring into the nursing requirements, and agreed to their suggestions that the inadequate nursing staff should be increased, and that a night nurse should be secured. Three guardians were of the Chairman's opinion, that they had been able to manage so well in the past that Sir Julian's suggestions were unnecessary, and "would land the board in expense"; but the other members had a larger sympathy for pain and poverty, and so Pembury paupers will be better cared for in sickness in the future, and their last hours will be made easier for them.

ECONOMY AT MACROOM.—The Guardians at Macroom are going to introduce nuns as nurses to their infirmary, and they intend to provide additional buildings for their accommodation. But the *Cork Herald* reports the Chairman, Father O'Connell, as announcing that "the nuns would not work later than nine o'clock at night," and what does that mean? Are the unhappy sick paupers to be left alone during the weary night watches? Can any intelligent guardians consent to such an unmerciful arrangement? A thrifty member said that he was quite certain the consumption of stimulants would be diminished after the introduction of the nuns, but he did not say by whom the consumption has taken place? If the paupers are to be cut short in their comforts they won't appreciate the change. The present Matron receives £40 per annum and rations, the Rev. Mother is to have £50 and no food. Is £10 per head the usual yearly grant for rations in Ireland? We cannot emulate that low rate of catering for our nurses in England.

CROYDON NURSING INSTITUTE. — The Croydon Guardian the other day contained a kindly letter from "One who feels indebted to Croydon nurses." The writer refers to their exertions during the influenza epidemic, and suggests that the nurses having now moved into a larger house, a few additional comforts might be presented to their new sitting-room as a slight token of grateful interest from former patients.

SOUTHMOLTON AND ITS NURSE ASSOCIATION. —A meeting was lately held in the Assembly Rooms, Southmolton, to consider the possibility of retaining the services of the nurse. A letter was read from the Secretary of Queen Victoria's Jubilee Institute, saying that if the Association decided to retain the nurse for another year, she should receive a larger salary and a considerable increase of work. It was finally resolved that the district should be enlarged by the addition of the parishes of Chittlehampton and Filleigh, and Lady Susan Fortescue and Lady Poltimore proposed a plan for increasing the salary to the suggested rate of £80 per annum. It is an encouragement to nurses to see their work so well appreciated, and the country districts thus bestirring themselves to avoid relinquishing the trained services which they have learnt to value.

NORTHAMPTON INSTITUTE FOR TRAINED NURSES.—The report of this association shows it to be arranged with considerable forethought with respect to the comfort, convenience, and varied incomes, of employers of trained nurses. The nurses themselves have also, we are glad to read, improved house accommodation, including a pleasant sitting-room. There is a special feature in this report which is of particular interest to all district nurses. We refer to "Rules of the Invalid Loan Society" and the list of articles which can be had on hire, and paid for in advance. This obviates many disadvantages attendant on nursing the poor, and those whose incomes do not allow of the purchase of such needful accessories as water-pillows, steam-kettles, crutches, towels, mackintoshes, &c. A capital list of sick necessaries is given, with the moderate charge for each, and we are delighted with the common sense which has formulated a simple scheme calculated not only to aid in the nursing, but to foster the self-respect of the borrowers.

SHORT ITEMS.—Merthyr Board of Guardians have decided to give their nurse at the Workhouse Infirmary the handsome salary of £20 per annum. We hope they did not expect many trained and certificated applicants to present themselves as candidates for the post! — Mrs. Bousfield is going to have a second annual sale of work at 248, Portsdown Road, Maida Vale, on October 22, in aid of the funds of the Invalid Children's Aid Association.—The Government of Bosnia has appointed lady doctors to attend women in all the principal towns; they will have free quarters provided for them, and a fixed yearly income from State resources.—Brechin Victoria Nursing Association has engaged a nurse, who will enter on her duties in the present month.—The Bridgend District Nursing Association has secured the services of an experienced nurse, who is also a good Welsh scholar. Miss Mainwaring had previously been working at the Welsh Branch of the Queen Victoria Jubilee Institute.—The Chard Board of Guardians have increased their nurse's salary from £20 to £25 per annum.—Miss Garnett-Clarke has opened a nursing home at Ellastone, Walton, Clevedon, for invalids of both sexes. The house has a lovely view, and the rooms are large and bright. Miss Clarke will be assisted by a trained nurse and masseuse.—Three trained nurses from the Q.V.J.I. are to nurse the sick poor "throughout the Parliamentary Borough" according to arrangements made by the Hampshire Nurses' Institute.

### Lectures for Asylum Attendants.

By William Harding, M.B.

### V.—FOOD.

The duties of the nurse with regard to the food of her patients fall into two divisions. In the first place, with respect to the great mass of those under her care who are able to take their meals in the common dining-room; and, secondly, there are those acute or special cases which require more individual attention.

The condition of a dining-room at meal times is a very good test of a woman's abilities as charge nurse, and many excellent nurses are not suited for that post. The comfort of the meals and the appearance of the viands depends very much upon the attention paid to the table appointments and the method of serving the food. The charge nurse must use her head as well as her hands. Table-cloths, where used, are a sad trial. A clean cloth put on for breakfast is often so soiled as to be hardly fit for use at dinner, and yet neither the resources of the ward stocks, nor the capabilities of the laundry, will allow of a fresh cloth for each meal. Everything should be made as appetising and as bright as possible. When most asylums are in the country, and wild flowers are common, there is no reason why in the quieter wards flowers should not be constantly present on the tables in the summer months. In addition to their brightening effect, the arrangement and gathering of them will give pleasure and light occupation to some of the inmates. It is needless to remark that knives and forks should be clean, and that salt-cellars and mustard pots should be kept freshly supplied. These things appear to be matters of course, but in practice they are curiously apt to be overlooked.

Meal times are occasions on which every patient can be individually noticed. Each nurse should have a table assigned to her, and to which she always attends. The same patients should always sit at that table, and in their usual seats. The nurse can then perceive at once either want of appetite or any alteration in the patient's condition. She will become acquainted with the peculiarities and habits at meal times of those at her table, *e.g.*, those who bolt their food without masticating it; those who eat slowly and require extra time; those who would steal from the plates of their neighbours, and, if permitted, gorge themselves to sickness. The class who bolt their food are liable to choke, owing to their greedy or stupid efforts to swallow large masses. Inability to take food, or any change in appearance, should be at once reported to the charge nurse. The latter will see to the serving and exercise a general supervision. Some patients cannot take the ordinary amount, and others will require, perhaps, more than an average helping. It is natural that those who do much work and are energetic will require more food than some of the others, but the nurse should be careful that the workers are not pampered at the expense of other patients too stupid to complain or look after themselves. It rests, to a great extent, with the charge nurse, whether, even in the noisiest wards, the dining-room is quiet and orderly or noisy and turbulent. The maintenance of order and decency at meals has a civilizing influence upon the worst cases, and grace before and after meat should not be slurred over, but looked upon as part of the regular discipline of the day. When a dining-room is entered unexpectedly, and the nurses are found to have left their tables and to be gathered together in knots gossiping, the probability is that the meals are badly served, and the patients, restless, quarrelsome, and unsettled.

The food should be served as rapidly as possible, in order that it may not become cold. In placing it upon the plates also, the nurse should remember that the appearance of the food often decides, in the case of a dainty appetite, whether it will be eaten or left almost untouched. A second course should not be served until the first is finished. All stages of dinner should not be going on at the same time. The quantity of fluid taken should be noticed, and as far as possible regulated within reasonable limits. The amount that some lunatics will imbibe is extraordinary. This at any time impairs the digestion, and at the evening meal in demented cases is of importance from another point of view.

*(To be continued.)*

### Nursing Homes.

### VII.—BIRMINGHAM WORKHOUSE INFIRMARY.

It is easy to reach this wonderful building by means of a steam tram which starts from a spot not far from the Town Hall. Of surpassing ugliness, these cars prove themselves such economists of our time, that we abstain from more criticisms on their unprepossessing looks, but we cannot refrain from a remark on the rate of their charges. On the day of our visit several women in pauper uniform were amongst our fellow travellers, and we were surprised at their alighting some time before the workhouse was reached; however, the remarks of other passengers soon solved the mystery, for they explained that the "penny stage" ended here, and if the poor old folks rode the rest of the way they would have to pay double. "I call it a reg'lar shame," said a kind-faced young girl. "You're right, Miss," replied a labouring man, "it ought to be a penny all the way for them poor things; anyhow, it's but a small bit of a ride for the money, and I'm sure nobody 'ud grudge it to them old folks, even if the rest of us had still to pay the 2d. It's cruel hard lines for they feeble creatures to have to climb up the hill all weathers, and get in punctual after any little outing they've took!" Truly, we may call this "a hard world," but in respect of sympathy for their poorer brethren, English workmen and workwomen are wont to agree with swift cordiality.

When the tram finally deposited us at a corner of the workhouse territory, we soon noticed the vast extent of ground occupied by the numerous blocks of buildings, and also the shrubberies, recreation and kitchen gardens, which spread over more land still, whilst the extent of sky and the supply of fresh air appear practically unlimited at this elevated spot.

The porter at the entrance gate gave immediate attention to our inquiries for the infirmary, and after noting down our names in an imposing-looking volume gave clear directions for our guidance, and we at once passed on, wondering anew at the extent of the accommodation needed for the housing of the destitute, whilst we frequently admired the present condition of gardens which, three years ago, were comparable only to a wilderness!

A few more formalities accomplished, and then we found ourselves entering the far-famed main corridor, which we felt was curiously impressive, partly, no doubt, from its unlikeness to all preconceived ideas, and partly also for its brightness and lightness, and its surprising perspective. The pretty pavement and the radiant cleanliness all culminating in one far distant spot of light, where a pleasant green lawn was dimly seen. As we walked on towards this goal we were amused to see a quiet young man coming by on a tricycle, and we learnt from our guide that he was one of the doctors, and that they all "went their rounds" in this fashion to save both time and fatigue. Perhaps this piece of information did more to impress us with the size of this infirmary than any other single fact. At present 1,500 beds are dedicated to the sick, acute as well as chronic cases. Epileptics are nursed in a separate block, and so are infectious diseases. The latter have also a little house for the use of the nurses, who work under a sister, and in all ways are quite apart from the rest of the staff.

On our way down the corridor we saw the fine dispensary, and the admirable kitchen, with all its modern appliances, which were intelligently explained to us by the head cook, who appeared very proud of her orderly domain.

We had a peep at the nurses' mess-room, and at various wards, all bright-looking and in beautiful order, with well-grown, healthy-looking plants to give thoughts of hope amidst the sorrows and sufferings of humanity.

Crossing the garden we reached the Nurses' Home, at the back of which a large tennis ground is specially devoted to their own use.

By the way, the outside public is apt to think probationers distinctly ill-used, where hospital rules do not countenance tennis playing; but where provision is made for this recreation, it is usually found that only a very limited number of the nurses care to indulge in it.

To return to the Nurses' Home, we find that a pleasant sitting-room, well-furnished, and rich in cosy chairs, is provided; whilst bath-rooms are conveniently placed, and passages are warmed by hot-water pipes—a wise precaution, for in winter this airy site must be a somewhat bleak one.

The bed-rooms are very pleasant indeed, and are nicely furnished and home-like, whilst the night nurses' accommodation is exactly the same, but situated on the top floor, quite quiet, and also distinctly separated from the day nurses' quarters. Altogether, there is very much to commend in this Home, and one gets a pleasant conviction of the progress made in infirmary nursing and in the type of workers; but we want more of the latter. Such an institution as this Birmingham one could train twice as many probationers, with not only benefit to themselves and to the patients, but to the public at large. The supervision being, happily, in the hands of experienced and thoroughly-trained ladies, there seems no reason why the materials at hand should not be utilised extensively. A while ago infirmary nursing was spoken of with a very general accent of contempt, not only on account of the quality of the attendants employed, but also because it was thought that "a lot of chronic cases" taught nothing to aspiring probationers. Now as regards medical nursing especially, we can assure our readers that a vast number of acute cases pass through infirmary wards, getting skilled treatment and making good recoveries. Again, many surgical and operation cases get into the wards, and *where the nursing is known to be efficient* the surgery is also very advanced, and the results are brilliant.

As for the chronic cases, which comprise rheumatism, heart, kidney, and lung diseases, besides paralysis and many forms of nerve troubles, can nothing be learnt from these? We are inclined to think that the woman who has successfully nursed these helpless cases, and learnt to wash and move them without pain to patient or strain to herself, and who has acquired an intelligent comprehension of the medical treatment as well as of the dietary; of the ventilation of wards, as well as the observation of fresh symptoms; this probationer, we maintain, has every probability of finding herself, at the end of her training, well-fitted to take her place in the foremost ranks of trained nurses.

That infirmary work is hard and incessant there can be little doubt, but the only way to reduce it to more reasonable limits is by increasing the staff, doubling or trebling the present numbers, and making the standard in all ways to correspond with those of the great general hospitals where probationers abound.

We must cordially congratulate this, the greatest of poor-law infirmaries, on its rapid and satisfactory progress, and on the excellent training school it has organised, and we trust that ere another three years have passed, at least another hundred nurses will be added to the nursing staff.

### Girl-Nurses.

"How young can I take up nursing?" is a far more frequent query than "At what age am I best fitted to begin my training?" The second question generally indicates a hopeful measure of capacity, whilst the first may mean something very different. "How young" is gradually merging into "the fittest age" at all the best general hospitals, and 25 years is usually accepted as best for the beginning of the probationer's training. Infirmaries at present take younger women, which is a pity, but they will probably raise their standard later on. The reason we say "it is a pity," is this, infirmary nursing is not lighter work than that in hospitals, and when trained women are the universal attendants in the first as they are in the last, it will be found necessary to make the same limit of age as experience has decreed best for the hospitals. Another very serious objection to the employment of such young girls in infirmaries, incurable or special hospitals, is the class of the cases there received. Technically known as "heavy cases" they embrace a vast variety of diseases, having helplessness as their one general characteristic. It is useless to say "a great many of these could be better nursed by men," for, in spite of the obviousness of the truth, the supply of thoroughly trained male nurses of good character is far too small to meet the difficulty at present. There are not sufficient opportunities for the necessary training of suitable men, as but few hospitals admit male probationers. As long as patients suffering from all kinds of nerve diseases—such as paralysis, for instance—have to be nursed by women, a great deal may well be said on the subject of age. In a general hospital ward it is only a very small proportion of men who are quite helpless, and therefore the senior nurse is accustomed to personally attend to the needs of her exceptionally bad cases. Moreover, as her probationers must be at least twenty-five, they may often reach an average of thirty years of age. Unfortunately in other institutions, where the majority of the patients are exceedingly helpless, the probationers are very young indeed. This ought not to be the case in any place where invalids exist whose hands and feet no longer serve them. Such chronic sufferers as these require many attentions which should only be demanded of experienced and mature women. At present this is not so. Witness the evidence of "supply and demand" embodied in a recent advertisement from a London Workhouse Infirmary, "Candidates must not be less than 21 years of age, well-educated, and have had at least one year's experience in nursing in some public institution." Other advertisements demand "young nurses," leaving the exact age at which they admit girls, to the imagination of the reader. We feel sure of sympathetic comprehension on the part of all trained nurses, and we hope ere long committees and boards will awaken to the impropriety of very young women being selected as attendants on those who have the misfortune to be "helpless" patients.

### Miss Meyrick's Nursing Hostel.

Miss Meyrick has taken 33, Beaumont Street, and made it into a very bright and pretty home for the reception of private patients. There are charming bedrooms with plenty of windows to let in light and show to the best advantage the dainty furniture and fittings. The bedsteads are excellent, and we think that medical and surgical patients ought to make rapid progress towards convalescence amidst such pleasant surroundings. Miss Meyrick has a famous list of patrons and friends, whose well-known names are a guarantee of the respect in which she is held in the nursing world. And we cordially wish her success in her new and cheerful hostel.

### Some Australian Experiences.

(*Continued from page* clxvii., *Vol. XII.*)

In the wards each bed is arranged to suit certain cases, all have mosquito nets, neatly held back in the day time by red braid fastened with a rosette. The ward furniture is good—white deal tables in the centre, scrubbed every morning, a chest of drawers for dressings, bandages, &c., large cupboards for linen, splints, &c., and an antiseptic box. Each patient has a locker, which contains comb, brush, and any little parcel; no food of any description is allowed in the wards after meal time, and as all meals are provided by the hospital, there is no "conglomeration" in the lockers such I have seen in England, where patients have to "find" tea, sugar, butter, and soap; this I consider a great stride in the right direction. Clothing of all kinds belonging to patients is kept in a rack away from the ward; dressing gowns and shirts are also provided by the hospital; in this way a considerable amount of unnecessary lumber is kept out of the ward. There is also a "poison cupboard,' kept under lock and key, and a trolley to take the "dressings" and lotions along the ward on. At each end of the ward is a basin stand, with hot and cold water laid on for the doctors to wash their hands at. Sir Alfred Roberts, whom I mention with deepest respect, left nothing wanting for the careful nursing of the sick and comfort of the nursing staff. The work is both good and varied; people travel from all parts of the Colonies to be treated there. Hydatids forms a very interesting feature, and all the usual surgical work. There is a group of cottages for isolation and suspicious cases at the far end of the grounds. The operation theatre is a fine specimen of completeness and convenience, electric light being laid on, and all the newest improvements and instruments. A cabinet, costing a thousand pounds, filled with every kind of instrument, was presented to the hospital. The physicians and surgeons always operate in white linen jackets, which, besides being so clean, give an air of thoroughness to their work.

It would astonish the folks who think the majority of medical men in the Colonies are men who failed at home, to see the group of skilled and efficient men who assemble to operate, or hear the splendid lecturers who taught us so much. All these men were anything but failures, but fully qualified men from "Bart's," "Guy's," University College, Edinburgh University, &c,, &c., and the success of their work proves their ability. It is not at all an unusual thing to see Sir Alfred Roberts at these operations; his interest in the place is unbounded. In my day the Medical Superintendent was Dr. Jenkins and the House Surgeon was Mr. R. Brinton, while the House Physician was Dr. Wallis, all from St. Bartholomew's.

In the "A" pavilion there is a beautiful chapel, with several stained-glass commemoration windows; there is a splendid organ given by Messrs. Paling and Co., Sydney, a trifle too large for the place perhaps. Service is held there every Sunday, at which the Matron, most of the nurses and sisters off duty, patients, and servants attend.

There is a large medical school in connection with the Hospital, and a number of students attend every morning to dress and otherwise see to patients.

The nursing staff is composed of bright, educated women, a trifle younger than we take them in England, but as they turn out very good nurses, their youth is in their favour. Each nurse's rank is denoted by her dress; there is no danger, for instance, of a probationer being mistaken for a sister, as probationers, assistant nurses, head nurses, and sisters all wear a different uniform indoors, but outdoor uniform is the same for all but the sisters. All, from the probationers upwards, get four hours off duty every day, afternoon and evening alternately, and a whole day once a fortnight, one of these being from six p.m. Saturday till ten p.m. Sunday, and permission to sleep at home if they like, the consequence is that there are no jaded, anxious, pale faces but the entire staff looks bright, cheerful, and contented. Salaries range from £18 to £40, which is the "sisters" salary; uniform is found. In addition to long hours off duty, there is so much for their amusement, such as tennis, "At Homes" in the Matron's room, pleasant little dances, at which may be seen officers from whichever gun boat happens to be in port, private theatricals, &c. Many of our naval officers have been nursed there, so the place is familiar to them all. The food, too, is very good indeed and plenty of it; always sausages, chops, steak or bacon for breakfast, a joint, two vegetables, and pudding for dinner; and cold meat, bread, butter, and jam for tea. The nurses dine at twelve a m., one of the sisters presiding, and at one p.m. the sisters dine. The diet scale for the patients is also good; in fact food is not stinted at all.

There is a great interest taken in the patients welfare by folks outside. Every Monday afternoon Miss Mollie Crook (since deceased, I regret to say) came with her little band of musical friends, as well as some Italians (whom she paid herself) with violins and harps and a very nice concert they made. Then Mrs. Canon Stephens and her daughter visited and read. Several ladies also formed a flower mission, and came twice a week with fresh flowers. On Saturday afternoon a Mr. Macarthy came to teach all boys up to sixteen or seventeen to make fancy things, work out puzzles and different games, and Lady Allen often gave a tea party, coming with her daughter to assist, so on the whole things were not at all dull for the patients; indeed, to my mind, they received considerable attention; it is just typical of the generous heart of the Australians. I found them ever thus. Then Christmas too, is another festivity all round, for although it is very hot and uncomfortable just then, everyone pounds away at decorations, and eats plum pudding and roast beef most religiously, and we would not have it otherwise either; it is such a bond with our dear old country. Of course at this time there are flowers in abundance, and among them a very pretty bush, something like Pink May; it is called "Christmas Bush," as it blooms just then and forms the chief decoration. This and gum tree are cut down and tied to the verandah posts all over the city, and look very effective. Then there is the Christmas tree, covered with lovely things; you almost wonder where they all come from, and when the excitement of lighting comes up it is difficult to feel fifteen thousand miles from England.

I mention all these details because I am so often asked, "Is it not very rough out there?" No, certainly not in the capitals or large towns, everything, on the contrary, is most comfortable, and the working classes are very much better off there, too. Of course, in the "bush" it is rough; so it is in the remote parts of England, Ireland, Scotland, and Wales.

Later on I will give you a "rough sketch," to the best of my ability, and leave you to choose where you would rather work.

During my stay at the "Prince Alfred" it was my good fortune to meet with people whom I hope to call dear friends to the end of my life.

(*To be continued.*)

### Presentation.

Miss Pollard, nurse-in-charge of the Cornwall Lunatic Asylum, Bodmin, was presented by her fellow nurses with a handsome travelling writing case on the occasion of her resignation. Miss Pollard is going to Colorado, where she is to be married very shortly.

### Everybody's Opinion.

*[Correspondence on all subjects is invited, but we cannot in any way be responsible for the opinions expressed by our correspondents. No communications can be entertained if the name and address of the correspondent is not given, or unless one side of the paper only be written on.]*

### VICTORIA JUBILEE INSTITUTE FOR NURSES, EDINBURGH.

"A Correspondent" writes: This Institute has progressed rapidly under Miss Guthrie Wright's energetic management. The house, 29, Castle Street, Edinburgh, is now entirely devoted to the work. It has been admirably organised by Miss Peter. The accommodation for nurses is good. District nurses have ample appliances in separate rooms for their work, and provision is made whereby the risk of infection is minimised. The work seems to be much appreciated by the people, it has been very considerably extended, and is still growing. We feel that the first President, the late Lady Rosebery, would have been much gratified could she have lived to see the present extent and efficiency of the work.

### BRASSEY HOME, VENTNOR.

"Nurse Sophie" writes: I felt I must write to you about the Brassey Home that has removed to Ventnor, Isle of Wight, from St. Leonards. I was amongst the first that went and found it most comfortable. It is situated in a very pretty part, and the air is very healthy, and there is a splendid view of the sea. It is also easy to get at any place of interest in the island. I thought this information might be useful to other nurses, who, like myself, are glad to get a pleasant holiday cheap. I have always found at seaside places the charges were exorbitant, whereas at Ventnor Home everything comes within the reach of a nurse's pocket.

### Notes and Queries.

To Correspondents.—1. Questions or answers may be written on post-cards. 2. Advertisements in disguise are inadmissible. 3. In answering a query please quote the number. 4. A private answer can only be sent in urgent cases, and then a stamped addressed envelope must be enclosed. 5. Every communication must be accompanied by the writer's full name and address, not necessarily for publication. 6. Correspondents are requested to help their fellow nurses by answering such queries as they can.

### Queries.

(1) *Midwifery.*—Is there any hospital where a nurse who cannot afford to pay, can be trained in midwifery?—*S. H.*

(2) *Hoary Locks.*—Is there any preparation which will bleach the hair without injury to it?—*S. H.*

(3) *Winter Climate.*—Information wanted,—*Egrotons.*

(4) *Cholera or Fever Nursing.*—Where to apply?—*Constance.*

(5) *Would-be Probationer.*—Is there any hospital where a young girl of 17 might be trained?—*Nippie.*

(6) *National Health.*—I should be very much obliged if you could give me the address of Secretary N. H. S.—*Nurse Ruth.*

(7) *Age.*—Can anyone inform me where a lady of nearly 50 can get trained?—*E. S.*

(8) *Would-be Probationer.*—Where can a housemaid with good references get trained? She is 24 years of age.—*Little Sister.*

(9) *Bournemouth.*—Can anyone recommend a convalescent home for children at Bournemouth?—*Trixey.*

### Answers.

(1) *Midwifery* (S.H.)—Apply to Mrs. Nichol, at 12, Buckingham Street, Strand, and she will kindly give information.

(2) *Hoary Locks* (S.H.)—Age is the only trustworthy bleacher for hair. We are sorry to find a nurse condescending to consider that art can improve upon nature in this respect.

(3) *Winter Climate* (*Egrotons*).—You must conform to our rules and send your name and address.

(4) *Cholera or Fever Nursing* (*Constance*).—It is a pity you did not take your sister's opinion before you troubled busy people to reply to your enquiries. Matrons of fever hospitals are too much engaged just now to have their time needlessly wasted.

(5) *Would-be Probationer* (*Nippie*).—Seventeen is much too young for a girl to begin training as a nurse. Do you mean nurse or ward-maid? You do not say. All information you require can be found in "How to Become a Nurse," by Honnor Morten, price 2s. 6d., at "Scientific Press," 140, Strand.

(6) *National Health* (*Nurse Ruth*).—Address Secretary, 53, Berners Street, London.

(7) *Age* (E. S.).—Apply to Matron, Royal Free Hospital, Gray's Inn Road; to Matron, Guy's Hospital; or Matron, London Hospital, for rules for Paying Probationers. Of course you could not go as a regular (non-paying) probationer into any hospital or infirmary.

(8) *Would-be Probationer* (*Little Sister*).—You are too young, 25 is the age to begin training. Read "How to become a Nurse," by Honnor Morten.

### Caldecote House Convalescent Hospital, Bushey Heath.

This little hospital has been doing steadily-increasing work since 1888, when it was started by Miss Derham and Miss Dunn for the reception of such surgical cases as the ordinary convalescent homes were unwilling or unable to admit. The first home contained but 10 beds, and now 33 patients can be admitted into the charming house which Mr. Hamer's liberality has enabled Miss Dunn to occupy. It is an ideal spot—a pretty house in a large garden in a fine situation, and the air is good enough to partly explain the miracles of healing which take place at Caldecote House. The children's appetites are really a constant surprise to the inexperienced visitor who knows how the ordinary sick child turns away from his food. At Bushey Heath they turn to it! The wards are so bright and light, with large windows through which the little people see the garden in bad weather, whilst on all good days they are carried out, beds and all, if necessary, to live in the pure air during all their waking hours. Hitherto this convalescent home has been a private enterprise, but now Miss Dunn has wisely obtained a committee to help her and to relieve her of her financial responsibilities. This will leave her free to devote her entire energies to the supervision of her patients and household. As a trained and experienced nurse, with a special aptitude for the management of children, Miss Dunn is especially fitted for the position of Lady Superintendent. She is also fully competent to train the paying probationers she receives into her pleasant home.

### Nurses' Bookshelf.

"Some Thoughts About Nursing"* is the title of a lecture given by Dr. Oswald Browne to the nurses of the Metropolitan Hospital, and now published in a pamphlet, which will be read with pleasure by all, and with profit by many nurses.

"A Primer of the Art of Massage"† (for learners), by Dr. Stretch Dowse. This tiny book could be conveniently carried in the pocket by any one of the learners for whom it is complied. It is excellently illustrated, and the subject is clearly put before the reader. We are told that "some individuals are utterly unfitted for the office" (of masseur or masseuse) "by nature, by education, by general development, and by disposition," and again, "whilst considering the masseur, I am anxious to draw your attention to several points of importance. The first is, that you keep yourselves in good health." This sound advice is followed by more details, and equally to the point, respecting the work and workers. The various movements are explained in a simple manner. The treatment of spinal curvature is specially referred to, and so is abdominal massage, which is, perhaps, the greatest difficulty of all to the beginner, in fact Dr. Stretch Dowse says, "It will take you months and months with daily practice to manipulate this region with any hope of being successful" . . . Therefore he inculcates gentleness and patience as well as perseverance on the part of his pupils.

* "Some Thoughts About Nursing," by Oswald Browne, M.A., B.A., M.R.C.P. (Publishers: Griffith, Farran, and Co., 39, Charing Cross Road.) Price Sixpence net.

† "Primer of the Art of Massage," by Dr. Stretch Dowse (Publishers: Simpkin, Marshall, Hamilton, Kent, and Co., London).

### Minor Appointments.

[We propose to insert from time to time under this heading the appointments of Assistant Matrons, Charge and Staff Nurses, if our readers will kindly keep us informed of their promotion.]

Three Counties Asylum, Hitchen.—Miss Ellen Taylor, who was trained at Cane Hill Asylum, has been made Assistant Matron at the Three Counties Asylum, and Miss Isabella Braines has also been appointed an Assistant Matron at the same asylum. Miss Braines received her training at the Three Counties Asylum.

Eastern Hospital, Homerton.—Miss Ada Carwardine, who was trained at the London Hospital, has been appointed Assistant Matron at Homerton.

### The Result of a Chill.

The Monition of Two Kinds of Fever *(continued)*.

As a rule the girls were more affectionate than the generality of sisters, and since their stay at Hollacombe they had lived in tender regard to each other's wellbeing. But now pecuniary worry was beginning to tell on Hilda's temper, and at times she hinted broadly that Sybil needed an agent to manage her affairs.

"It is a failure of the Ruthvens to let people profit by their ingenuousness," she continued. "Didn't poor father lose two hundred pounds by foolishly backing a bill? Aren't we invariably cheated by dressmakers? We must keep our wits about us if we are to live by them."

In this parental strain she discoursed for a quarter of an hour, until Sybil rose at a pause, and, with a sigh, put on her hat, and went out of the house. She had felt of late a little hurt at Hilda's acerbity. It was not seemly for a younger sister to lecture her, and to charge her with incompetence to conduct her business. But most of all she was offended with the continual slurs which Hilda cast upon the engaging visitor. She could not think of him as a common swindler. There was honesty in every line of his face, and the slightly lingering pressure of his strong hand at parting had convinced her that he wished her well. For the first time for many months she felt dissatisfied and wretched. It was late October, and the Moor was searched with a biting wind that came howling from the east. She made for the summit of Kestor, holding her hat upon her ruffled brown tresses, and leaning to the wind that pierced her. Down in the valley the Teign was rushing, but the gale quenched its roar with exultant huzzas as it sped by the granite mounds on the hill-top. Mile on mile of purple and yellow undulations spread around her. Cawsand, like a portly parent, smiled on its children the lesser tors; below, the smoke of Chagford was lost in the clear air, and the rooks were tossing on the brow of Middleton. All was fair, glowing, inspiring, but she had lost for once her interest in the scene. Had she stayed indoors she might have wrangled with Hilda and marred the calm of their happy hermitage. It was best to come out, she argued. But the wind was stinging and unfriendly, as if in mockery of her sudden mood of despondency. There was no repose in the midst of the booming. In a few minutes she descended through the brakes, and saw Hollacombe in deep shadow. The house looked so grim and dreary, set there between dark hills, that she wondered whether the stranger had spoken in sarcasm when he praised it. Well, they had been happy there, and she had often wondered how long the charm of the place would hold. To-day she felt acute loneliness, and had a sharp yearning for society. As for art, it was as sordid as any other mundane thing. An ideal was beautiful enough, as long as one had a full purse, but when money was lacking for long, she defied the veriest enthusiast to worship. If Hilda had not given way at this first pinch, affairs might have adjusted themselves in a week or two; but it was impossible to work serenely and endure the inevitable reproaches for the failure of her quixotic scheme. Besides, Hilda had no right to dictate to her. There seemed now no remedy but to yield up all hope, sell the furniture, and go back to London in search of situations as governesses. What an ignoble termination to an idyl! She went down the slope, half resolved to sound Hilda upon the advisability of leaving Hollacombe.

In a bedroom on a Regent's Park flat a nurse was reading by the fire while her patient dozed, hidden by the hangings of an antique bedstead. His inspirations were more regular than they had been for many days, and to-night the thermometer showed an encouraging decrease of temperature. The gas was low, and the nurse strained her eyes in the flickering firelight to read her senses to wakefulness with a novel. As she pored over the pages the sound of midnight pleasure-traffic found its way to the stillness of the sick-room. Now and again the clatter of hoofs and the grinding of wheels ceased abruptly, a cab door was shut with a slam, and a chattering of people returned from the play was heard on the steps of the neighbouring houses. At last the vast city slept, and the dull clang of church clocks told each quarter of the small hours to the heavy-pacing constable below.

Nurse Parkinson was hopeful of her present patient's case. He had passed through the severest stages of rheumatic fever with uncommon patience, though he had exhibited the usual resentment of his sex when the doctor forbade him to leave his bed at the first favourable change in his condition. A few relatives and many friends had called from time to time, and a litter of visiting cards lay upon the table by the bedside. There were letters, too, on the mantelpiece, which he had read when he felt well enough. Some contained inquiries concerning his illness, others were business communications, and one roll was printer's proofs sent for correction three weeks ago. It was clear to Nurse Parkinson that her patient was a person of some distinction, for most of the daily papers had recorded his illness; but until she was sent to attend him she had never heard of him by name. She at once "took a fancy to him"; she wrote to a friend, because he bore up so bravely in his pains, and only fretted at the thought of being idle and helpless. Such amiability as his was scarce among invalids with rheumatic pangs shooting all about their bodies; in short, he was a delightful case, and it was a true pleasure to wait upon him.

It was just after seven when the bed-curtain was pulled aside, and the patient looked out with a wideawake gaze.

"I have had a remarkably good sleep, nurse," he observed. "What's the time? Seven! Why, I've been asleep for ten hours. I'm practically well, and decidedly hungry."

"That's right," she said, rising to prepare breakfast.

The invalid stretched his arms, and clasped his hands under his head as the daylight fell on his pillow. It was plain to him that he was convalescent, and a placid smile settled upon his face.

"Not a single ache now," he murmured, gratefully. "I shall insist on being allowed to get up."

"Not until the doctor has seen you," said Nurse Parkinson. "We can't allow a relapse, Mr. Lancaster."

"The postman," he said, as a knocker sounded in the street. "That reminds me I have a score of letters to answer. I'll attend to some of them at once."

The nurse descended for the letters, and returned with a couple, and a flat parcel in brown paper.

*(To be continued.)*

### Appointments.

[It is requested that successful candidates will send a copy of their applications and testimonials, with date of election, to The Editor, The Lodge, Porchester Square, W.]

Three Counties Asylum, Hitchen, Herts.—Miss L. H. Treddell has been appointed Matron of the Three Counties Asylum, Hitchen. Miss Treddell was trained at the County Asylum, Lancaster.

### Wants and Workers.

A home wanted for a healthy girl of 17, able to work. Has been greatly neglected and needs firm and kind supervision. Could assist with children or in any domestic capacity under the immediate direction of a responsible lady. Address Mrs. Arnold, Main Street, Barton-under-Needwood.

